# Avoidance and Inhibition Do Not Predict Nonrespondent Bias Among Patients With Inflammatory Bowel Disease

**DOI:** 10.2188/jea.JE20100087

**Published:** 2011-01-05

**Authors:** Rafael J. A. Cámara, Stefan Begré, Roland von Känel

**Affiliations:** Department of General Internal Medicine, Division of Psychosomatic Medicine, Inselspital, Bern University Hospital, Bern, Switzerland

**Keywords:** refusal to participate, behavior and behavior mechanisms, bias (epidemiology)

## Abstract

**Background:**

It has been suggested that participant withdrawal from studies can bias estimates. However, this is only possible when withdrawers and nonwithdrawers differ in an important way. We tested the hypothesis that withdrawers are more likely than nonwithdrawers to be avoidant and negatively affected.

**Methods:**

A total of 1160 participants with inflammatory bowel disease were recruited at different sites in Switzerland. Their levels of avoidance coping and negative affectivity were rated by means of 2 short baseline questionnaires. One year later, they were sent a longer follow-up questionnaire. The primary outcome was return versus non-return of the follow-up questionnaire within 3 months. After controlling for potential confounders identified in an extensive literature search, we estimated the odds of returning the follow-up questionnaire for 1 standard deviation of avoidance coping and negative affectivity.

**Results:**

The odds ratio for 1 standard deviation was 1.03 (95% confidence interval: 0.89–1.18) for avoidance coping and 1.02 (0.89–1.17) for negative affectivity.

**Conclusions:**

The odds of returning the questionnaires did not depend on avoidance coping or negative affectivity.

## INTRODUCTION

Clinical researchers regularly face the problem of a significant number of patients refusing to participate in a study or subsequently failing to return a questionnaire containing important information. In randomized clinical trials with a reasonable sample size, adequate blinding and concealment, and intention to treat analysis, these difficulties may limit the generalizability of findings. In observational research, however, if nonresponse is selective, ie, nonrespondents are not just a random sample of the sample, then estimates might be biased.^1^

The term “nonrespondent bias” (also referred to as volunteer bias, nonresponse bias [also used with different meanings], or nonparticipation bias) was first used in 1979 in a classic paper describing 9 potential biases of interest,^2^ and usage of this term has increased over time.^3^ A clear divergence of nonrespondents from respondents is a necessary condition for nonrespondent bias.^1^ Given the high frequency and proportion of nonrespondents in studies, efforts have been made to find out if such divergences exist and, if so, whether they are associated with certain characteristics. Several demographic parameters have been thoroughly investigated.^4^^–^^8^ However, data on personality characteristics that potentially influence nonrespondent bias are scarce.

In this study, we examined 2 novel personality characteristics—avoidance coping and negative affectivity. In avoidant coping, individuals react to a demanding situation by distraction or social diversion, particularly if the situation constitutes a difficulty that must be solved.^9^ This strategy may be useful in moderation: gaining distance from an obstacle by participating in social activities may help in more easily finding a solution later on. However, extensive avoidance coping may result in unresolved situations. Highly avoidant patients might attempt to escape from anything suggestive of their illness and might thus be more prone to postpone completion of a questionnaire.

Negative affectivity implies that given situations or experiences are more likely to provoke negative emotions and a negative perception of those situations. Negative affectivity is associated with irritability, anxiety, depression, and general mental distress. Individuals who are negatively affected have unfavorable opinions of themselves and other people.^10^ Negatively affected persons receiving a questionnaire, for example, might be more likely to consider possible burdens and needed time and less likely to believe in personal and social benefits. Additional reasons for assuming that avoidant coping and negative affectivity might be involved in nonresponse are that both have been shown to be related to diminished medication adherence.^11^^,^^12^

We hypothesized that people with chronic diseases who show evidence of avoidance-oriented coping and/or a tendency to experience negative affect might be less likely to respond to a questionnaire. Confirming one or both of these hypotheses would suggest an elevated risk of bias in cases of personality-related exposures or outcomes (eg, subjective outcome measures), which would necessitate the assessment of personality characteristics and the comparison of outcomes in respondents and nonrespondents. Conversely, a rejection of both hypotheses, with sufficient statistical power, would indicate a low risk of selective bias related to personality or coping strategies. In both cases, however, it would be necessary to further investigate whether common personality characteristics are possible contributors of nonresponse (eg, overcommitment to work may decrease nonresponse). We would like to emphasize that this study was not conducted merely to test if some other findings of our group might be biased by nonresponse. Our main objective was to find out if nonresponse is associated with avoidance behavior and negative affectivity, in order to provide important information for future studies of exposures or outcomes that might be related to these personality characteristics.

## METHODS

### Setting, design, and patients

Several studies have observed a higher response rate among individuals with a chronic condition, as compared with those without such a condition.^5^^,^^13^ Thus, we elected to examine psychological factors of nonresponse in a sample of participants who had the same chronic condition, ie, inflammatory bowel disease. In addition, emerging evidence suggests that this disease is associated with psychological factors.^14^ We therefore used data from a consecutive sample of adults with recurrent inflammatory bowel disease diagnosed according to the Lennard-Jones criteria.^15^ The data were collected from July 2006 through February 2008 by collaborators of the Swiss Inflammatory Bowel Disease Cohort Study in university hospitals, regional hospitals, and private practices in the Swiss cities of Basel, Bern, Geneva, Lausanne, St Gallen, and Zurich.^16^

The ethical committees of all the study sites approved the study protocol, and the research was conducted in accordance with the Declaration of Helsinki of 1975. Once patients had provided written informed consent, we assessed avoidance coping and negative affectivity (the main predictors) by means of case report forms containing 2 self-rating scales, as outlined below.^9^^,^^10^ On the same case report forms, patients provided information on age, sex, marital status, education, employment, alcohol consumption, and smoking. One year after enrollment we sent them a longer questionnaire, which was sent directly to the participants by post. Those who did not return the questionnaire within 3 months were defined as nonrespondents to follow-up.

Because both rejection and confirmation of our hypothesis would provide important information to researchers, we were particularly concerned to adequately power our study. We enrolled 1150 participants, which yielded a power of 95% to detect an odds ratio (OR) for 1 standard deviation (SD) of 1.33 with projected proportions of 20% nonrespondents at baseline and 20% nonrespondents to follow-up and a 2-tailed α-level of 0.05.^17^

### Examined predictors

To measure avoidance coping, we used the Task-Oriented Coping Scale of the Coping Inventory for Stressful Situations.^9^ Eight items are rated on a 5-point Likert-scale from “not at all” (1) to “very much” (5). The total value is equal to the mean of the items, and a minimum of 7 valid items is needed to compute a valid mean. To assess negative affectivity, we used the 7-item Negative Affectivity Subscale of the Type D Scale-14 because of its well-documented characteristics.^10^ Items are rated on a 5-point Likert-scale from false (0) to true (4), and the scores are summed. The total score ranges from 0 to 28. Up to 2 missing items can be replaced with the mean of the valid items, without significantly affecting the properties of the scale.

In the present study, the German and French versions had a variance of item means of 0.16 and a variance of item variances of 0.03, indicating good weighting of the Task-Oriented Coping Scale. Considering the brevity of the scale, a Cronbach’s α of 0.79 indicated good overall reliability. The Negative Affectivity Subscale had even better quality measures: a variance of item means of 0.13, a variance of item variances of 0.05, and a Cronbach’s α of 0.88. A shared variance of less than 1% showed very good distinction between the 2 personality questionnaires.

Since it has been demonstrated that avoidance coping and negative affectivity, as measured with the above described instruments, are consistent over time (test–retest reliability: 0.68^9^ and 0.72, respectively),^10^ we decided to wait a maximum of 9 months for questionnaires to be returned. In addition, every 3 months, we reminded late respondents at baseline to return the questionnaires, in order to minimize baseline nonresponse.

### Outcomes

One year after enrollment, the patients received a longer follow-up questionnaire assessing disease-related quality of life.^18^ Quality of life is a main exposure and a main outcome measure of several hypotheses of the Swiss Inflammatory Bowel Disease Cohort Study.^16^ However, the focus of the present study, as explained in the introduction, was the associations of avoidance coping and negative affectivity with nonresponse. We used a quality of life questionnaire because the probability of nonresponse to follow-up for such a questionnaire is high (a long questionnaire sent at 1-year intervals increases the risk of questionnaire fatigue). This makes nonresponse to follow-up easier to examine. The primary outcome was whether this questionnaire was returned within 3 months. Because most clinical trials and cohort studies are performed under time constraints, 3 months is a representative waiting period. However, one could argue that this period is arbitrary. In addition, this study of nonresponse to follow-up could have been biased by nonresponse at baseline. It was not possible to compute the relation between the examined predictors (avoidance behavior and negative affectivity) and nonresponse at baseline, because these predictors were assessed in the baseline questionnaire. Therefore, as a proxy for this relation, we examined the relation between the suspected predictors and late response at baseline (ie, the time needed to respond to the baseline questionnaires, in 1-month units). To test whether this proxy (ie, the continuum of the resistance model) was satisfactory,^7^ we also analyzed the relation between late response at baseline and nonresponse to follow-up.

### Control variables

Although nonresponse is considered an important potential source of selection bias,^1^^–^^3^ little is known of the mechanisms underlying response and nonresponse. Age,^6^^,^^13^^,^^19^ gender,^6^^,^^13^^,^^19^^,^^20^ marital status,^6^^,^^21^ educational level,^5^^,^^6^^,^^13^^,^^19^^,^^20^ and employment^13^ have often been reported as possible predictors of nonresponse or late response. Moreover, alcohol consumption^22^^,^^23^ and smoking status^2^^,^^23^ are believed to play a role in response to surveys on alcohol consumption and smoking. Because we found no external evidence to suggest that nonresponse is different among patients with Crohn’s disease, ulcerative colitis, and indeterminate colitis, we did not control for different forms of inflammatory bowel disease. However, for interested readers, we report the proportions of patients with these disease forms.

### Data analysis

First, we presented our sample and identified respondents and nonrespondents to follow-up. Second, we estimated changes in the odds of response to follow-up (primary outcome) as a function of avoidance coping and negative affectivity and computed the linear relation between both avoidance coping and negative affectivity and time to baseline questionnaire return (late response at baseline). We used SPSS 15 for Windows (Chicago, IL, USA) for these analyses.

Continuous variables were described by using means and SD; categorical variables were described by percentages and absolute values. Respondents and nonrespondents to follow-up were compared by using mean or percentage differences. For all differences, we supplied asymptotic 95% confidence intervals (CIs) and 2-sided *P* values, using the Mann-Whitney U test for questionnaire scores, the independent *t* test for age, and Fisher’s exact test for sex, marital status, education, employment, alcohol consumption, and smoking. Because time to baseline questionnaires return was very skewed, we used the Mann-Whitney U test despite the large sample size.

In a first model (model 1), we estimated the OR for 1 SD of avoidance coping, while controlling for all described control variables. The dependent variable was the odds of returning the follow-up questionnaire. In a second model (model 2), the same procedure was used for negative affectivity. For avoidance coping, negative affectivity, and age, the ORs referred to the factor by which 1 standard deviation multiplied the odds of returning the questionnaires; for sex, marital status, and smoking, they indicated the odds for females divided by that for males, the odds for married individuals divided by that for unmarried individuals, and the odds for smokers divided by that for nonsmokers, respectively. For the variables of education, employment status, and alcohol, the OR referred to the odds of belonging to a category compared with not belonging to that category.

In a third model (model 3), we computed linear regression coefficients with time to return of baseline questionnaire as the dependent variable and avoidance coping plus control variables as independent variables. The same was done with negative affectivity as the main independent variable (model 4). For avoidance coping, negative affectivity, and age, coefficients indicated additional months needed for 1 standard deviation; for sex, marital status, and smoking, coefficients showed the average difference in months between women and men, married and unmarried people, and smokers and nonsmokers, respectively; for education, employment status, and alcohol, they showed the difference between those who did and did not belong to the respective category (eg, fulltime employment vs no fulltime employment). All ORs and linear relationships were provided with their corresponding 95% CIs and 2-sided *P* values. The significance level was 0.05.

## RESULTS

### Descriptive results

Of the 1149 enrolled patients, 1% died or left Switzerland (mean age ± SD, 54.7 ± 19.4 years; proportion of women, 63.6%; proportion of patients with Crohn’s disease, 72.7%), and 15.9% returned no baseline questionnaire (ie, questionnaire-level nonrespondents) or an insufficiently completed baseline questionnaire (ie, item-level nonrespondents) (mean age ± SD, 38.7 ± 14.4 years; proportion of women, 45.3%; proportion of patients with Crohn’s disease, 64.8%). As compared with respondents at baseline, the participants who died or left Switzerland were 12.4 years older on average (95% CI, 3.8–21.0 years; *P* = 0.005), had a 12.1% higher proportion of women (−17.7% to 41.9%; *P* = 0.549), and a 15.1% higher proportion of patients with Crohn’s disease (−14.3% to 44.5%; *P* = 0.373). Nonrespondents at baseline were on average 3.6 years younger (1.3–5.9 years; *P* = 0.002), had a 6.2% lower proportion of women (−1.7% to 14.1%; *P* = 0.146), and a 7.2% higher proportion of patients with Crohn’s disease (0.6%–15.0%; *P* = 0.071). For nonrespondents at baseline, there was no information on marital status, employment status, education, or alcohol intake, because this information was assessed with avoidance behavior and negative affectivity in the baseline questionnaire. The Figure summarizes the selection of the 955 participants for analysis.

**Figure.**
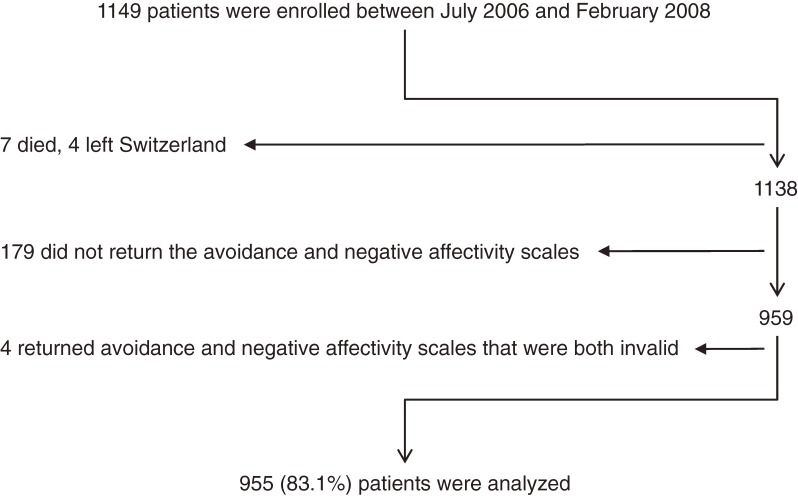
Flowchart of Patients Selected for Analysis. The Figure shows the process for selecting participants for analysis. Of the 959 remaining patients, 916 answered all items on the avoidance scale and 917 did so for the negative affectivity scale. Of the 43 partially completed avoidance scales, 26 were valid because only 1 item was missing. Of the 42 partially completed negative affectivity scales, 36 could be completed by replacing the unanswered or invalid items with the mean score for the valid items. Seventeen avoidance scales and 6 negative affectivity scales were unusable. We excluded 4 patients who returned avoidance and negative affectivity scales that were both unusable.

Of the 955 analyzed patients, 594 (62.2%) returned the follow-up questionnaire within 3 months and 361 (37.8%) did not. On average, participants who did not return the follow-up questionnaire were 1.52 months later in returning the baseline questionnaires. Apart from the time span until baseline questionnaires were returned, smoking was the only characteristic that distinguished the patients who returned the follow-up questionnaire from those who did not (Table 1).

**Table 1.** Characteristics of respondents and nonrespondents to follow-up

Characteristic	Total (955)	Respondents (594)	Nonrespondents (361)	Difference (95% CI)	*P*
Avoidance coping score	1.94 ± 0.82	1.96 ± 0.80	1.92 ± 0.84	0.04 (−0.07 to 0.14)	0.510
Negative affectivity score	10.33 ± 6.32	10.31 ± 6.27	10.36 ± 6.42	−0.05 (−0.87 to 0.78)	0.951
Time to baseline response (months)	1.91 ± 2.82	1.36 ± 1.69	2.88 ± 3.94	−1.52 (−2.01 to −1.03)	<0.001
Age (years)	42.29 ± 14.40	42.63 ± 14.21	41.74 ± 14.72	0.89 (−0.90 to 2.77)	0.356
Female sex	51.5% (492)	52.4% (311)	50.1% (181)	2.3% (−4.3% to 8.8%)	0.548
Married	49.2% (470)	51.3% (305)	45.7% (165)	5.6% (−0.9% to 12.2%)	0.095
Education					
ISCED 5A and 6	13.1% (124)	13.1% (77)	13.2% (47)	−0.1% (−4.6% to 4.3%)	1.000
ISCED 5B	17.6% (166)	17.1% (101)	18.3% (65)	−1.2% (−6.2% to 3.9%)	0.660
ISCED 3 and 4	67.2% (634)	67.6% (398)	66.5% (236)	1.1% (−5.1% to 7.3%)	0.775
ISCED 1 and 2	2.1% (20)	2.2% (13)	2.0% (7)	0.2% (−1.6% to 2.1%)	1.000
Employment status					
Full-time	57.2% (545)	56.4% (334)	58.6% (211)	−0.8% (−8.7% to 4.3%)	0.543
Part-time	22.9% (218)	24.3% (144)	20.6% (74)	3.7% (−1.7% to 9.2%)	0.203
Not working	19.9% (189)	19.3% (114)	20.8% (75)	−1.5% (−6.9% to 3.7%)	0.559
Alcohol consumption					
Daily	8.3% (78)	8.1% (48)	8.5% (30)	−0.4% (−4.0% to 3.3%)	0.903
Weekly	33.5% (316)	33.6% (198)	33.2% (118)	0.4% (−5.9% to 6.6%)	0.943
≤Monthly	58.3% (550)	58.2% (343)	58.3% (207)	−0.1% (−6.6% to 6.4%)	1.000
Smoking	30.8% (294)	27.4% (162)	36.6% (132)	−10.8% (−15.3% to −3.1%)	0.003
Diagnosis					
Crohn’s disease	57.6% (550)	56.9% (338)	58.7% (212)	−1.8% (−4.6% to 8.3%)	0.590
Ulcerative colitis	39.7% (379)	40.6% (241)	38.2% (138)	2.4% (−8.7% to 4.1%)	0.496
Indeterminate colitis	2.7% (26)	2.5% (15)	3.1% (11)	−0.6% (−1.7% to 2.7%)	0.684

Quantitative variables are presented as mean ± standard deviation, and qualitative variables as percentages (absolute numbers). There were 955 (100%) valid responses for age, sex, marital status, and diagnosis; 953 (99.8%) for negative affectivity and smoking; 952 (99.7%) for employment status; and 944 (98.8%) for education and alcohol consumption.

Abbreviations: CI, confidence interval; ISCED, international standard classification of education (www.uis.unesco.org; last access September 16, 2010): levels 1 and 2 are obligatory in Switzerland, and levels 3 and 4 allow access to 4-year higher education (level 5B) and university study (levels 5A and 6).

### Main results

The odds of returning the follow-up questionnaire (models 1 and 2 in Table 2) showed no relevant or significant logit-linear dependence from avoidance coping (OR for 1 SD, 1.03; 95% CI, 0.89–1.18) or negative affectivity (1.02; 0.89–1.17).

**Table 2.** Relation between suspected predictive factors and nonresponse to follow-up on logistic regression analysis

Variable	Model 1		Model 2	
	
	Odds ratio (95% CI)	*P*	Odds ratio (95% CI)	*P*
Avoidance coping	1.03 (0.89–1.18)	0.379	—	—
Negative affectivity	—	—	1.02 (0.89–1.17)	0.742
Age	1.04 (0.87–1.24)	0.652	1.06 (0.89–1.26)	0.523
Female sex	1.09 (0.81–1.46)	0.572	1.11 (0.84–1.67)	0.310
Married	1.16 (0.86–1.57)	0.335	1.12 (0.84–1.67)	0.310
Education				
ISCED 5A and 6	0.85 (0.32–2.25)	0.746	0.79 (0.30–2.07)	0.632
ISCED 5B	0.77 (0.27–2.16)	0.626	0.74 (0.27–2.04)	0.558
ISCED 3 and 4	0.80 (0.28–2.26)	0.675	0.76 (0.27–2.11)	0.594
Employment status				
Full-time	1.27 (0.80–2.00)	0.310	1.29 (0.82–2.03)	0.279
Part-time	1.13 (0.75–1.71)	0.553	1.15 (0.76–1.74)	0.503
Alcohol consumption				
Daily	1.04 (0.77–1.40)	0.800	1.04 (0.77–1.40)	0.786
Weekly	1.02 (0.60–1.72)	0.964	1.03 (0.61–1.74)	0.916
Smoking	0.67 (0.50–0.89)	0.007	0.65 (0.49–0.88)	0.004

Model 1 included 926 cases (97.0%) and examined the hypothesis that individuals with avoidant behavior are less likely to return a follow-up questionnaire; model 2 included 934 cases (97.8%) and tested the hypothesis that individuals with negatively affectivity might be less likely to do so.

Abbreviations: CI, confidence interval; ISCED, international standard classification of education (for additional information please refer to the legend for Table 1).

### Secondary results

Each additional SD (= 2.82 months) needed to return the baseline questionnaires decreased the odds of returning the follow-up questionnaire by 1.76 times (95% CI, 1.48–2.09; *P* < 0.001). Because there were no data on avoidance coping and negative affectivity for nonrespondents at baseline, we used late response at baseline as an approximation for nonresponse at baseline (Table 3). Time to return of baseline questionnaire (models 3 and 4 in Table 3) showed no relevant or significant linear dependence from avoidance coping (linear increase for 1 SD, 0.15 months; 95% CI, 0.05–0.36) or negative affectivity (0.06 months; 0.14–0.26).

**Table 3.** Relation between suspected predictive factors and late response at baseline on linear regression analysis

Variable	Model 3		Model 4	
	
	Coefficient (95% CI)	*P*	Coefficient (95% CI)	*P*
Avoidance coping	0.15 (−0.05 to 0.36)	0.139	—	—
Negative affectivity	—	—	0.06 (−0.14 to 0.26)	0.556
Age	−0.27 (−0.53 to −0.00)	0.048	−0.24 (−0.50 to 0.03)	0.080
Female sex	−0.59 (−1.02 to −0.15)	0.009	−0.52 (−0.95 to −0.08)	0.020
Married	−0.31 (−0.76 to 0.14)	0.180	−0.35 (−0.80 to 0.10)	0.129
Education				
ISCED 5A and 6	1.74 (0.27 to 3.22)	0.020	1.43 (−0.01 to 2.88)	0.051
ISCED 5B	1.34 (−0.12 to 2.80)	0.072	1.06 (−0.37 to 2.48)	0.146
ISCED 3 and 4	1.23 (−0.15 to 2.61)	0.082	0.91 (−0.43 to 2.26)	0.181
Employment status				
Full-time	−0.59 (0.84 to 1.67)	0.058	−0.56 (−1.17 to 0.05)	0.072
Part-time	0.03 (0.84 to 1.67)	0.034	0.03 (−0.65 to 0.70)	0.936
Alcohol consumption				
Daily	−0.29 (−1.11 to 0.52)	0.477	−0.30 (−1.11 to 0.51)	0.474
Weekly	−0.48 (−0.92 to −0.03)	0.034	−0.47 (−0.91 to −0.03)	0.038
Smoking	0.32 (−0.11 to 0.76)	0.145	0.26 (−0.18 to 0.69)	0.249

Models 3 and 4 investigated if time to baseline questionnaire response was associated with avoidance coping and negative affectivity, respectively.

Abbreviations: CI, confidence interval; ISCED, international standard classification of education (for additional information please refer to the legend for Table 1).

## DISCUSSION

In a meta-analysis of response rates for 68 internet-based surveys, the average ± SD was 39.6 ± 19.6%.^24^ Providing overall estimates for participation rates in randomized clinical trials and cohort studies, or response rates for questionnaire studies, is difficult because rates vary greatly. Most researchers will agree, however, that response rates below 60% are not unusual and that 80% is very good.^24^ For this reason, it is essential for clinical research to investigate if nonresponse is accidental and, if not, whether it is systematically associated with particular characteristics to a relevant degree. Conclusive data are lacking, and methodologists have focused almost exclusively on common demographic parameters: younger age^7^^,^^19^ and male gender^4^^,^^19^ predicted later responses. Other studies performed in different parts of the world found that younger age,^6^^,^^13^ male gender,^6^^,^^13^ being single or divorced,^6^^,^^8^^,^^21^ and a lower educational level^5^^,^^6^^,^^13^ were associated with reduced participation. In some studies, however, younger age^25^ and male gender^26^ were related with higher response rates. Predictors of nonresponse are often associated with the study topic^22^^,^^23^: in a study of alcohol consumption, interestingly, abstainers were less likely to respond than moderate drinkers.^22^ Researchers also investigated nonresponse at the item level^27^: amongst participants who returned questionnaires, they examined whether participants were more likely to skip some items rather than others. The patterns revealed were similar to those for nonresponse at the questionnaire level.^27^ However, most demographic differences between respondents, nonrespondents, and late respondents were small.^4^^,^^6^^,^^13^^,^^19^^,^^22^^,^^27^

Our results confirmed those earlier findings. Apart from smoking, which decreased the odds of returning the follow-up questionnaires by 1.5 times (95% CI, 1.1–2.0), the effects we found among our control variables were not relevant. Individuals needed to be 14.4 years (= 1 SD) older in order to return the baseline questionnaires 1 week earlier (*P* = 0.048). Men, on average, returned questionnaires 3 weeks later than did women (*P* = 0.009). These values are insufficient to bias estimates. If a systematic difference between respondents and nonrespondents to follow-up does indeed exist, other characteristics must be responsible for it.

Patients were asked in an open-ended question about their motives for participating in cardiovascular trials.^28^ The top answers were personal health benefits (82.2%), interest in research (44.1%), and the possibility of benefiting society (29.1%). Although everybody might be interested in benefiting themselves and society, not everyone would agree regarding the usefulness of studies. But what explains the difference? As it is not explained by educational attainment—only small^5^^,^^6^ or no^21^ differences have been found—a reasonable approach is to examine if personality plays a role. However, only a few studies have considered this possibility. A family study found that siblings of nonrespondents scored higher on scales of anxious depression and neuroticism; however, the siblings themselves were actually respondents.^29^ Data from a longitudinal study showed that unemotional behaviors and having a parent with an antisocial personality were predictive of contact difficulties, and that attention deficit hyperactivity disorder was predictive of refusals.^30^ A study on successful aging reported that those who consented to participate were less depressed but, interestingly, also perceived less social support.^31^ In an attempt to obtain information on the personality of nonrespondents at baseline, questionnaires were emailed to individuals who had revealed sufficient personal information on the web to rate their personality. The rating was performed by university students who were blinded to the outcome (ie, response versus nonresponse to the questionnaire). Nonrespondents were judged to be less agreeable and less open to experience.^32^ However, individuals who share private information in such a manner might not be representative in terms of personality.

We found no systematic difference between respondents and nonrespondents to follow-up with respect to avoidance coping and negative affectivity. Moreover, avoidance coping and negative affectivity did not delay the time to questionnaire response at baseline. It should be emphasized in this context that although late response at baseline and nonresponse to follow-up are not the same phenomenon, they are closely related, given that an increase of 2.8 months in response at baseline was associated with an almost 1.8-fold odds of nonresponse. However, this was insufficient to use late response as an approximation of nonresponse, a finding which conformed to those of previous studies.^7^^,^^33^ We conclude that studies of nonresponse should define a cut-off.

Are the present results reliable? Due to the fact that the rate of nonresponse to follow-up was higher than expected, the statistical power of the present study was not 95%, as we had planned, but 99%. Thus, even very small effects were statistically significant, and additional statistical power was not necessary. With respect to bias in the present estimates, the most important weakness of this study was that represented by the studied topic. We investigated potential relationships between personality characteristics (avoidance behavior and negative affectivity) and nonresponse; however, we could not assess those characteristics in the 183 (15.9%) nonrespondents at baseline (see Figure legend). We attempted to overcome this limitation by using late response at baseline as a proxy for nonresponse at baseline. As mentioned above, the association between late response at baseline and nonresponse to follow-up was insufficient for the use of late response as a proxy for nonresponse. Two hypotheses emerged from this observation. First, some patients may never respond, regardless of the time they are allotted. Second, the relation between response at baseline and response to follow-up could be weak, which, if true, suggests that nonresponse is not consistent within one person (this would diminish the risk of systematic differences between respondents and nonrespondents). We achieved a good baseline response rate (83.1%) and our outcome of interest was not rare (37.8% nonresponse to follow-up). However, our estimates could still be biased if nonrespondents do indeed differ systematically from respondents, which is as yet unproven. Specifically, we found that, on average, nonrespondents at baseline were 3.6 years younger than respondents. This difference is somewhat more pronounced than the age effect for late response to follow-up, but still small.

Given that bias is a systematic deviation from the truth and not a random deviation,^1^ nonrespondents can only bias estimates if at least one relevant characteristic systematically divides respondents and nonrespondents. Until such a characteristic is identified, there is insufficient evidence that nonrespondents bias estimates, and it remains reasonable to assume that nonresponse is random. This does not mean, however, that outcome estimates will necessarily be the same for nonrespondents and respondents. For example, a study found that, despite equally distributed smoking habits, respiratory symptoms, and lung function, the outcome—hospital admissions during follow-up—was twice as high in nonrespondents (9.9%) as in respondents (5.0%). The authors concluded that estimates were biased by nonresponse.^34^ We computed exact 95% CIs for the estimates of this study using STATA 11 for Windows and found that the estimate for the whole sample (5.6%, *n* = 1070) was within the 95% CIs for both the nonrespondents (5.5%–16.0%, *n* = 142) and the respondents (3.6%–6.6%, *n* = 928). If estimates vary randomly, ie, not because of bias, the true value will stay within the 95% CI in 95% of cases.^35^ We conclude that the difference between nonrespondents and respondents in that study can be explained by random variance alone. We nevertheless agree with the principal conclusion of the authors, that equal distribution of baseline characteristics is not sufficient to exclude nonrespondent bias.^34^ Research must continue to move forward and analyze more than common baseline characteristics.

Meta-analytic methods might be useful in distinguishing random differences from biases. In an email survey of 2127 clinicians, nonrespondents received as many as 5 email reminders and, if necessary, a sixth by fax. The outcome was the prescribing of contraindicated medications. Subgroups were defined by number of reminders needed. The estimate for the total group was within the 95% CIs of all 7 subgroups. An I^2^ statistic of zero indicated that there was no inconsistency among groups, other than random differences.^36^

To summarize, our findings show that avoidance coping and negative affectivity are unlikely to differ among respondents and nonrespondents to a questionnaire survey. In addition, our survey of the literature findings revealed no decisive factors underlying nonresponse. Further study of this topic is important because nonresponse is very frequent.
